# Efficacy and safety of combining low-dose methadone with ongoing opioid treatment for uncontrolled cancer pain: an open-label single-arm study

**DOI:** 10.1093/oncolo/oyaf215

**Published:** 2025-07-17

**Authors:** Takaaki Hasegawa, Toru Okuyama, Nana Suzuki, Yosuke Furukawa, Yoshihiko Tasaki, Moeko Iida, Asako Ito, Megumi Uchida, Yosuke Kubota, Shino Kikuchi, Hideo Yamakawa, Yoshihiko Harada, Tatsuo Akechi

**Affiliations:** Center for Psycho-oncology and Palliative Care, Nagoya City University Hospital, Nagoya 467-8601, Japan; Center for Psycho-oncology and Palliative Care, Nagoya City University Hospital, Nagoya 467-8601, Japan; Department of Psychiatry and Cognitive-Behavioral Medicine, Nagoya City University Graduate School of Medical Sciences, Nagoya 467-8601, Japan; Department of Psychiatry, Nagoya City University West Medical Center, Nagoya 462-8508, Japan; Center for Psycho-oncology and Palliative Care, Nagoya City University West Medical Center, Nagoya 462-8508, Japan; Center for Psycho-oncology and Palliative Care, Nagoya City University Hospital, Nagoya 467-8601, Japan; Center for Psycho-oncology and Palliative Care, Nagoya City University West Medical Center, Nagoya 462-8508, Japan; Center for Psycho-oncology and Palliative Care, Nagoya City University Hospital, Nagoya 467-8601, Japan; Department of Nursing, Nagoya City University Hospital, Nagoya 467-8601, Japan; Center for Psycho-oncology and Palliative Care, Nagoya City University Hospital, Nagoya 467-8601, Japan; Department of Clinical Pharmaceutics, Nagoya City University Graduate School of Medical Sciences, Nagoya 467-8601, Japan; Center for Psycho-oncology and Palliative Care, Nagoya City University Hospital, Nagoya 467-8601, Japan; Department of Clinical Pharmaceutics, Nagoya City University Graduate School of Medical Sciences, Nagoya 467-8601, Japan; Center for Psycho-oncology and Palliative Care, Nagoya City University Hospital, Nagoya 467-8601, Japan; Department of Nursing, Nagoya City University Hospital, Nagoya 467-8601, Japan; Center for Psycho-oncology and Palliative Care, Nagoya City University Hospital, Nagoya 467-8601, Japan; Department of Psychiatry and Cognitive-Behavioral Medicine, Nagoya City University Graduate School of Medical Sciences, Nagoya 467-8601, Japan; Center for Psycho-oncology and Palliative Care, Nagoya City University Hospital, Nagoya 467-8601, Japan; Department of Psychiatry and Cognitive-Behavioral Medicine, Nagoya City University Graduate School of Medical Sciences, Nagoya 467-8601, Japan; Center for Psycho-oncology and Palliative Care, Nagoya City University Hospital, Nagoya 467-8601, Japan; Center for Psycho-oncology and Palliative Care, Nagoya City University Hospital, Nagoya 467-8601, Japan; Center for Psycho-oncology and Palliative Care, Nagoya City University Hospital, Nagoya 467-8601, Japan; Department of Psychiatry and Cognitive-Behavioral Medicine, Nagoya City University Graduate School of Medical Sciences, Nagoya 467-8601, Japan; Center for Psycho-oncology and Palliative Care, Nagoya City University Hospital, Nagoya 467-8601, Japan; Department of Psychiatry and Cognitive-Behavioral Medicine, Nagoya City University Graduate School of Medical Sciences, Nagoya 467-8601, Japan

**Keywords:** methadone, neoplasms, pain, palliative care, opioid

## Abstract

**Background:**

Pharmacological options for refractory cancer pain are limited. This study aimed to investigate the efficacy and safety of the combined use of low-dose methadone and ongoing opioid treatment for uncontrolled cancer pain.

**Methods:**

This was a prospective, open-label study. Participants were patients with uncontrolled cancer pain despite dose titration of opioids. Patients received low-dose methadone (starting dose of 5 or 10 mg/day) combined with another ongoing opioid therapy. The primary outcome was the proportion of responders (defined as ≥33% reduction in average pain intensity on the numerical rating scale [NRS]) on day 15. Pain intensity and adverse events according to the Patient-Reported Outcome Common Terminology Criteria for Adverse Events were evaluated at baseline, on days 8 and 15.

**Results:**

Nineteen patients participated in this study, 11 (57.9%) of whom had neuropathic pain. The mean daily oral morphine equivalent dose before combination was 112.6 mg. The primary outcome occurred in 13 (68.4%) of patients (95% CI, 43.4 to 87.4). The mean average NRS was 5.9 at baseline, which decreased significantly to 4.2 and 3.3 on days 8 and 15 (*P* < .001), respectively. The worst pain intensity on NRS decreased significantly over time. Adverse effects, including nausea, vomiting, constipation, and somnolence, which were new or had worsened from baseline, were reported in 26.3%, 26.3%, 5.3%, and 26.3%, respectively. Delirium was observed in one patient.

**Conclusion:**

Low-dose methadone with ongoing opioid treatment shows potential efficacy in the management of uncontrolled pain with acceptable adverse events.

**ClinicalTrials.gov identifier:**

UMIN000038924

Lessons learned

- In this prospective open-label study, we determined the efficacy and safety of low-dose methadone as an add-on to ongoing opioid therapy for patients with uncontrolled cancer pain.
- During 15-day study periods, 68.4% participants were categorized as responders, with ≥33% pain reduction; the mean reduction in the average numerical rating score was 2.6 points.
- Although more research on combination strategies of methadone with another opioid is needed, this study describes a promising algorithm for introducing low-dose methadone as an add-on to another ongoing opioid therapy that has been demonstrated to be safe and effective during the follow-up period.

**Trial information**

**Table:** 

Trial information	
**Disease**	Refractory cancer pain, regardless of conventional opioid therapy
**Stage of disease/Treatment**	Advanced or recurrent
**Prior therapy**	Adequate dose titration and current treatment with oral morphine equivalent of 60 mg/day or more
**Type of study**	Prospective, single-arm observational study
**Primary endpoints**	Proportion of pain reduction from baseline
**Secondary endpoints**	Pain intensity, frequency of daily breakthrough opioid use, and safety
**Additional details of endpoints or study design** The primary endpoint was the percentage of patients with ≥33% average pain reduction from baseline. The proportion of pain reduction was calculated using the following formula: (pain intensity at baseline − pain intensity on day 15)/(pain intensity at baseline) × 100. Single institution, single arm study	

**Drug information**

**Table:** 

Drug information	
**Generic/Working name**	Methadone
**Company name**	TEIKOKU SEIYAKU
**Drug type**	Analgesic
**Drug class**	Opioid
**Dose**	5 or 10 mg/day
**Route**	Oral
**Schedule of administration** Oral methadone has been introduced as an add-on medication to ongoing opioid therapy. After day 7, the physicians increased the methadone dose. Scheduled intervention: see Figure 1.	

**Dose table**

**Table:** 

Dose table			
	Dose of drug	Number enrolled	Number evaluable for toxicity
Ongoing opioids with oral morphine equivalents less than 120 mg/day	5 mg/day	12	12
Ongoing opioids with oral morphine equivalents 120 mg/day or more	10 mg/day	7	7

**Patient characteristics**

**Table:** 

Patient characteristics	
Age	
median (IQR), years	69 (55-71)
range, years	39-75
Sex	
Female	8 (42.1%)
Primary cancer	
Gastrointestinal	6 (31.6%)
Head and neck	6 (31.6%)
Breast	2 (10.5%)
Multiple myeloma	2 (10.5%)
Lung	1 (5.3%)
Prostate	1 (5.3%)
Unknown	1 (5.3%)
Tumor stage	
Advanced or recurrent	19 (100%)
Comorbidity of heart disease	
Yes	1 (5.3%)
Comorbidity of lung disease	
Yes	2 (10.5%)
History of gastrointestinal surgery	
Yes	3 (15.8%)
Performance status (ECOG)	
1	1 (5.3%)
2	10 (52.6%)
3	7 (36.8%)
4	1 (5.3%)
Pain^a^	
Neuropathic pain	11 (57.9%)
Multiple bone metastasis	9 (47.4%)
Spinal cord invasion	2 (10.5%)
Cancer skin ulcer	2 (10.5%)
Visceral pain	2 (10.5%)
Pleural invasion	1 (5.3%)
Duration of opioid therapy	
Within 1 month	4 (21.1%)
1-3 months	5 (26.3%)
3-6 months	2 (10.5%)
More than 6 months	8 (42.1%)
Ongoing opioid therapy	
Oxycodone	9 (47.4%)
Morphine	5 (26.3%)
Fentanyl	5 (26.3%)
Background oral morphine equivalent daily doses (mg)	
Median (IQR)	90 (80-120)
Range	60-216
Concomitant medication^a^	
Nonsteroidal anti-inflammatory drugs	13 (68.4%)
Acetaminophen	8 (42.1%)
Anticonvulsants (pregabalin, all)	8 (42.1%)
Steroids	2 (10.5%)
Antidepressants	1 (5.3%)

a Multiple-choice responses were available.

Abbreviations: ECOG, Eastern Cooperative Oncology Group; IQR, interquartile range.

**Table:** 

Primary assessment method	
**Title**	The proportion of responders (defined as ≥33% reduction in average pain intensity on the numerical rating scale [NRS]) on day 15
**Number of patients screened**	27
**Number of patients enrolled**	19
**Number of patients evaluable for toxicity**	19
**Number of patients evaluated for efficacy**	19
**Evaluation method**	The proportion of pain reduction was calculated using the following formula: (pain intensity at baseline − pain intensity on day 15)/(pain intensity at baseline) × 100.
**Outcome notes** Response assessment: see Table 1.	

**Adverse events (AEs)**

**Table:** 

Adverse events								
	Baseline			During study period			New or worse than at baseline	
	Grade 1-2	Grade 3	Grade 4	Grade 1-2	Grade 3	Grade 4	≥1 Grade	≥2 Grade
Nausea	8 (42.2%)	3 (15.8%)	1 (5.3%)	7 (36.8%)	4 (21.1%)	0 (0%)	5 (26.3%)	2 (10.5%)
Vomiting	6 (31.6%)	0 (0%)	1 (5.3%)	9 (46.8%)	0 (0%)	0 (0%)	5 (26.3%)	2 (10.5%)
Constipation	12 (63.2%)	2 (10.5%)	0 (0%)	8 (42.1%)	2 (10.5%)	0 (0%)	1 (5.3%)	0 (0%)
Somnolence	16 (84.2%)	3 (15.8%)	0 (0%)	16 (84.2%)	2 (10.5%)	1 (5.3%)	5 (26.3%)	0 (0%)

Adverse events were evaluated using the Patient-Reported Outcome (PRO) Common Terminology Criteria for Adverse Events (CTCAE) at baseline, on days 8 and 15.

## Discussion

In this prospective open-label study of patients with advanced cancer, we assessed the efficacy and safety of oral methadone in combination with ongoing opioid treatment. Few studies have investigated the combination of low-dose methadone for refractory cancer pain^1–3^; moreover, to our knowledge, this is the first study to use a pre-planned approach to assess the efficacy and safety of low-dose methadone as a co-analgesic to another ongoing opioid therapy.

In this study, 68.4% participants were categorized as responders, with ≥33% pain reduction; the mean reduction in the average numerical rating score was 2.6 points. These findings are consistent with those of previous retrospective studies examining the use of low-dose methadone as a coanalgesic^1–3^ and a prospective study of opioid switching to methadone as a second-line opioid.^4^ In addition, the analgesic effects in this study appeared to be greater than those of switching to another opioid.^5^^,^^6^ To date, switching to opioids other than methadone is generally recommended for refractory cancer pain, despite titrated opioid doses^7–9^; however, low-dose methadone combined with other opioids might also be an option. In addition, because conversion ratios between methadone and other opioids have not been definitively established, there is a concern that opioid switching to methadone may increase pain^10^; however, few patients on methadone combination therapy in the present study experienced worsening cancer pain.

Furthermore, adverse events in the present study were measured using the PRO-CTCAE, and the most common new adverse events or those that were worse than baseline were nausea/vomiting and somnolence. The safety of methadone has been questioned, and careful consideration when switching from opioids to methadone is essential.^11^ Intensive monitoring of sedation caused methadone to be administered when it was titrated. Adverse events were also evaluated during intensive monitoring. With the exception of 5 cases in which methadone could not be continued due to difficulty in taking oral medication owing to tumor progression, no patients discontinued methadone treatment because of adverse effects. Accordingly, the combined use of low-dose methadone and ongoing opioid therapy appears to be safe. Two patients experienced QTc prolongation > 500 ms. Therefore, if low-dose methadone is initiated, caution should be exercised with the combined use of QTc-prolonging drugs, which are frequently used in palliative medicine and medical oncology.

This study has several limitations that should be considered. First, it was a prospective cohort study and did not include a comparison group. The observed reduction in pain intensity may be due to spontaneous improvement, regression to the mean, or obsequiousness bias. Therefore, the efficacy of the combined use of low-dose methadone and ongoing opioid treatment revealed in this study should be confirmed in randomized controlled trials. Second, this study was conducted at a single institution, and thus the findings cannot necessarily be generalized to other situations, such as other populations or clinical settings. Third, the participants did not receive high-dose opioids at baseline, and the appropriate dose of methadone for combination use remains unknown (especially in oral morphine equivalents of 300 mg/day or more). Fourth, the small sample size may have led to an underestimation of serious adverse events.

## Background

Cancer pain is a common symptom that negatively affects the quality of life of patients with cancer.^12^ Several clinical guidelines for cancer pain have shown that the use of strong opioids (morphine, oxycodone, fentanyl, or hydromorphone) is the mainstream approach to alleviate cancer pain.^7–9^ Although opioids generally provide an analgesic effect in the majority of patients, a certain proportion of patients experience refractory pain despite adequate use of opioids.^13–16^

Pharmacological options for refractory cancer pain are limited and consist of opioid switching and adjuvant analgesics, such as gabapentinoids.^17^^,^^18^ However, these analgesics are often unsuccessful, and the development of effective analgesic treatments for refractory cancer pain is urgently needed. Among these, the use of methadone as a part of opioid switching is controversial.^11^ Methadone has several unique profiles, not just as a synthetic mu-opioid agonist, but also as an *N*-methyl-d-aspartate receptor antagonist.^19^ The conversion ratios from other opioids have not been well established, and there is a concern that switching to methadone may increase pain. Although its analgesic effects are promising,^4^ the safety of switching to methadone has been a growing concern.^10^^,^^11^ Owing to the long and unpredictable half-life of methadone, there are concerns regarding impaired consciousness and respiratory depression associated with overdose, forcing methadone initiation to be introduced under adequate monitoring.

A recent focus has been on induction with low-dose methadone in combination with conventional opioids.^1–3^ It is presumed that less exacerbation of pain will occur after introducing methadone with the continued use of ongoing opioids by not completely switching from other opioids to methadone. However, no prospective studies have been conducted, and the findings are insufficient. Therefore, we designed a single-arm study to investigate the analgesic efficacy and safety of low-dose methadone combination therapy in patients with cancer pain and inadequate analgesic effects, regardless of opioid therapy.

## Methods

### Study design

This was a prospective, single-arm observational study of patients with advanced cancer, including those with solid tumors and hematological malignancies, at Nagoya City University Hospital. Consecutive patients who had follow-up appointments with a palliative care center (including an outpatient palliative care clinic and an inpatient consultation team) at Nagoya City University Hospital were enrolled from February 2020 to August 2022. Both outpatients and inpatients were included in the study. This study was approved by the Institutional Review Board and Ethics Committee of Nagoya City University Graduate School of Medical Science and was conducted in accordance with the principles of the Declaration of Helsinki. All participants were provided with a detailed description of the purpose and methods of the study before completing the questionnaire. Written informed consent was obtained from all the participants. The study was registered with the University Hospital Medical Information Network (UMIN000038924).

### Patients

Inclusion criteria were as follows: (a) diagnosis of any cancer type including hematological malignancy, (b) age 20-75 years, (c) pain related to cancer, (d) average pain intensity ≥4 on a 0-10 Numerical Rating Scale in the last 24 hours, (e) adequate dose titration and current treatment with oral morphine equivalent of 60 mg/day or more, and (f) ability to take oral medication. The exclusion criteria were as follows: (a) disturbed consciousness, (b) cognitive impairment, (c) undergoing or preplanned concurrent radiotherapy, (d) first-line chemotherapy started 7 days before enrollment, (e) QTc prolongation (male >470 ms, female >480 ms), and (f) contraindications for methadone.

### Study treatments

**Figure 1.**
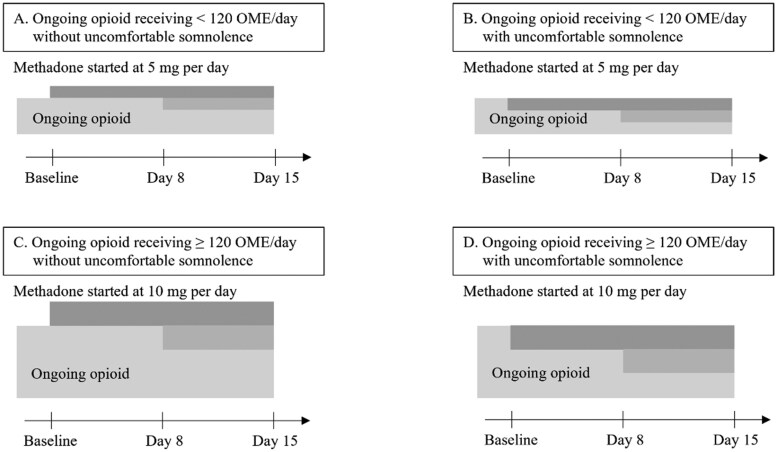
Scheduled intervention: combined use of low-dose methadone with ongoing opioid treatment. In patients receiving ongoing opioids with oral morphine equivalents <120 mg/day, oral methadone was started at a dose of 5 mg/day. In patients with ongoing opioids receiving oral morphine equivalents ≥120 mg/day, oral methadone was started at a dose of 10 mg/day. In patients who experienced uncomfortable somnolence before combination therapy, the ongoing opioid therapy dose was reduced to 20 mg oral morphine equivalents per 5 mg methadone. After day 7, the physician increased the dose of methadone. The patients who reported satisfactory analgesia continued to receive the same dose of methadone. Ongoing opioid and methadone use can be decreased during the study period. Abbreviation: OME, oral morphine equivalent.

In patients receiving ongoing opioids with oral morphine equivalents less than 120 mg/day, oral methadone was started at a dose of 5 mg/day. In patients with ongoing opioids receiving oral morphine equivalents ≥ 120 mg/day, oral methadone was started at a dose of 10 mg/day. Oral methadone has been introduced as an add-on medication to ongoing opioid therapy. In patients who experienced uncomfortable somnolence before combination therapy, the ongoing opioid therapy dose was reduced by 20 mg of oral morphine equivalents per 5 mg of methadone (methadone was started as a partial opioid switch). The use of low-dose methadone and ongoing opioid therapy is shown in Figure 1.

On day 8, the patients who reported satisfactory analgesia continued to receive the same dose of methadone. After day 7, the physicians increased the methadone dose. Ongoing opioid or methadone use was reduced in patients with uncomfortable somnolence during the study period. Adjuvant drugs could also be added by the physicians. The study period was 15 days.

### Measurement

All patients reported pain intensity (both average pain and worst pain) over the last 24 hours, number of breakthrough opioids used, and the Patient-Reported Outcome (PRO)—Common Terminology Criteria for Adverse Events v.1.0, Japan Clinical Oncology Group version (CTCAE) at baseline and on days 8 and 15.^20^

The primary endpoint was the percentage of patients with ≥33% average pain reduction from baseline.^5^^,^^21^ The proportion of pain reduction was calculated using the following formula: (pain intensity at baseline − pain intensity on day 15)/(pain intensity at baseline) × 100. The secondary endpoints included the percentage of pain reduction (≥33% reduction in the worst pain from baseline, ≥50% reduction in average from baseline, and ≥50 reduction in the worst pain from baseline), pain intensity over the last 24 hours on days 8 and 15 (both average pain and worst pain), frequency of daily breakthrough opioid use, and adverse events. Adverse events, including nausea, vomiting, constipation, and somnolence, were assessed using PRO-CTCAE. Delirium was assessed using the Diagnostic and Statistical Manual of Mental Disorders, 5th edition, when the patient developed disturbance of consciousness during the study period.

### Data collection

At the baseline assessment, patient characteristics, including age, sex, primary cancer site, clinical stage, comorbidity, Eastern Cooperative Oncology Group performance status, and laboratory tests (serum bilirubin, aspartate aminotransferase, alanine aminotransferase, creatinine, calcium, potassium, magnesium, and QTc interval^22^) were assessed from the medical records. Pain intensity, frequency of daily rescue use, and adverse events, including QTc prolongation, were assessed at baseline, on days 8 and 15. The mechanism of cancer pain was assessed by a palliative care physician (Diplomate, Specialty Board of Palliative Medicine, Japanese Society for Palliative Medicine), and neuropathic pain was diagnosed based on the International Association for the Study of Pain algorithm. Opioids, nonsteroidal anti-inflammatory drugs, acetaminophen, and other co-analgesics (such as anticonvulsants, antidepressants, and steroids) were also recorded at baseline, on days 8 and 15. Continued methadone use was assessed on days 8, 15, and 29.

### Sample size calculation

Opioid switching is a standard strategy for refractory pain despite adequate opioid dose titration.^7–9^ Based on previous studies,^5^ we assumed that the response rate (≥33% reduction in average pain) to opioid switching for refractory pain would be approximately 40%. In this study, we set the threshold for the primary endpoint as the percentage of patients with ≥33% reduction in average pain from baseline at 40%. Assuming a set expected value of 70%, 16 patients were required in this study according to the exact binomial test (2-sided α  =  0.1, 1 − β  =  0.8). Considering patient ineligibility, a sample size of 19 patients was used.

### Statistical analysis

Efficacy and adverse event analyses were performed on the intent-to-treat dataset, which included patients who received at least one methadone dose. The last observation-carried-forward method was used to impute missing and dropout data, including pain scores and adverse events, and the last observed non-missing value was used to fill in missing values at a later point in the study. We also adopted a complete case analysis for the sensitivity analysis to investigate the stability of the results.

To investigate pain reduction after the intervention, we conducted repeated-measures analyses of variance for all measures. Statistical significance was set at *P* < .05. Statistical analyses were performed using SPSS v.28.0 (IBM Corp., Armonk, NY, US).

## Results

### Characteristics

**Figure 2.**
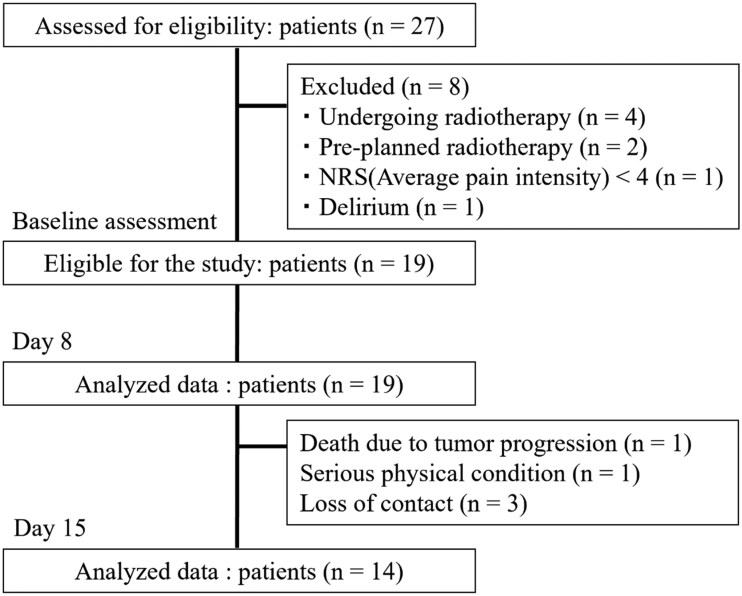
Flow chart of participants and follow-up. Abbreviation: NRS, numerical rating scale.

From February 2020 through August 2022, 27 patients received a combination of low-dose methadone and an ongoing opioid treatment. Of these 27 patients, 8 patients were excluded for the following reasons: undergoing radiotherapy (*n* = 4), pre-planned radiotherapy (*n* = 2), NRS (average pain intensity) < 4 (*n* = 1), and delirium (*n* = 1). Of the remaining 19 patients, all of whom were eligible and agreed to participate. All participated in the follow-up survey on day 8, and 14 participated on day 15 (Figure 2). The median overall survival was 66 days (interquartile range, 52-206 days). During the 15-day study period, one participant died on day 14 because of cancer progression. The causes of pain were variable and included neuropathic pain, multiple bone metastases, cancerous skin ulcers, spinal cord infiltration, and visceral pain.

Before combination therapy, the following strong opioids were used: oxycodone in 9 patients (47.4%), morphine in 5 patients (26.3%), and fentanyl in 5 patients (26.3%). The mean oral morphine equivalent before combination therapy was 112.6 mg (standard deviation [SD], 44.8 mg), ranging from 60  to  216 mg (median, 90 mg; interquartile range [IQR], 80-120 mg).

The initial methadone dose was 5 mg in 12 patients (63.2%) and 10 mg in 7 (36.8%). Fourteen (73.7%) patients continued methadone treatment at the end of study period. On day 15, the methadone dose was 5 mg in 4 patients (21.1%), 10 mg in 6 (31.6%), 15 mg in 1 (5.3%), and 20 mg in 3 (15.8%).

At baseline, co-analgesics included nonsteroidal anti-inflammatory drugs in 13 patients (68.4%), acetaminophen in 8 (42.1%), anticonvulsants in 8 (42.1%), and corticosteroids in 2 (10.5%).

The average number of breakthrough opioids use at baseline was 2.7 times per day, ranging from 0 to 10 (IQR, 1-4).

### Response rate and pain intensity

On day 15, ≥33% reduction in average pain from baseline was achieved in 13 (68.4%; 95% CI, 43.4 - 87.4)—the lower limit of the 95% CI exceeded the 40% threshold. In sensitivity analysis using complete cases, pain reduction of 33% or more from baseline (average pain) was achieved in 10 (71.4%; 95% CI, 41.9-91.6) (Table 1).

**Table 1.** Percentage of patients with a ≥ 33% or ≥50% reduction in average and worst pain from baseline on days 8 and 15.

Definition of response	≥33% pain reduction	≥50% pain reduction
	Percentage (95% CI)	Percentage (95% CI)
Average pain		
Day 8	42.1 (20.3-66.5)	26.3 (9.1-51.2)
Day 15	68.4 (43.4-87.4)	52.6 (28.9-75.6)
Worst pain		
Day 8	26.3 (9.1-51.2)	15.8 (3.4-39.6)
Day 15	36.8 (16.3-61.6)	21.1 (6.1-45.6)

Abbreviation: CI, confidence interval.

**Figure 3.**
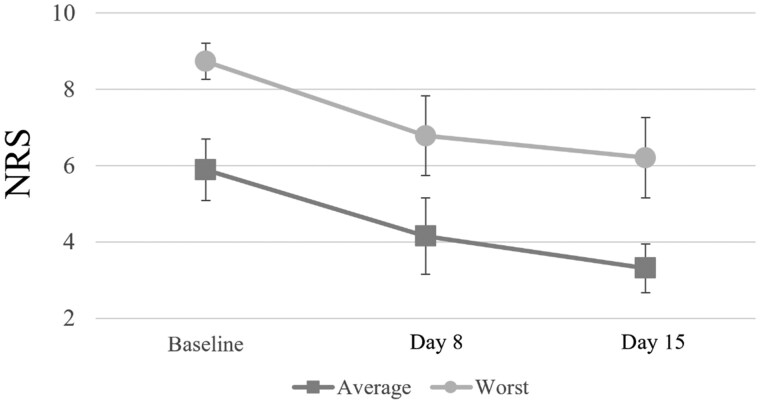
Mean average pain intensity and worst pain intensity during the study period. Abbreviation: NRS, numerical rating scale.

Regarding pain intensity (Figure 3), the mean average pain intensity significantly decreased from 5.9 (SD 1.8) at baseline to 4.2 (2.2) on day 8 and 3.3 (1.4) on day 15 (*P* < .001), and the mean worst pain intensity significantly decreased from 8.7 (1.0) at baseline to 6.8 (2.3) on day 8 and 6.2 (2.3) on day 15 (*P* < .001). The mean change in average pain intensity on day 15 was 2.6 (1.4); the mean change in worst pain intensity on day 15 was 2.5 (2.1). During the 15-day study, no patient experienced worse average pain, and only one patient experienced an exacerbation of the worst pain.

### Adverse events

The most common opioid-related side adverse effect before combination therapy was somnolence (100%). Nausea, vomiting, constipation, and somnolence that was new or had worsened from baseline were reported in 26.3%, 26.3%, 5.3%, and 26.3% of patients, respectively. One patient reported Grade 4 somnolence, but there were no limitations in activities of daily living.

QTc prolongation was observed in 2 patients. In 1 male patient, the QTc was 507 msec on day 14. In addition to methadone 20 mg/day, levofloxacin 500 mg/day and trazodone 75 mg/day were administered. Methadone was continued at 20 mg/day, levofloxacin was discontinued, and the trazodone dose was reduced, resulting in a QTc of 420 ms. In 1 female patient, the QTc was 532 msec on day 13. In addition to methadone 20 mg/day, levofloxacin 250 mg/day and fluconazole 50 mg/day were administered. Discontinuation of levofloxacin and fluconazole was not possible, and the methadone dose was reduced to 15 mg/day, resulting in a QTc of 425 ms. No QTc prolongation was observed in other patients. Delirium was observed in one patient. Besides methadone, a direct risk factor for delirium is tumor brain invasion.

### Co-intervention

The average number of breakthrough opioids use was 2.3 times (0-7 times) per day on day 8 and 2.4 times (0-6 times) per day on day 15.

The mean oral morphine equivalents of other combination therapy were 87.7 mg (SD, 25.6 mg), ranging from 60 to 120 mg (median, 90 mg; IQR, 60-120 mg) on day 8, 66.3 mg (SD, 28.8 mg), ranging from 30 to 120 mg (median, 60 mg; IQR, 46-90 mg) on day 15.

No patient received new co-analgesics other than an opioid during the study period. Furthermore, no patients received radiotherapy during the study period. Of the 19 patients, 11 received ongoing anticancer treatment during the study period. Only one patient with multiple myeloma had an anti-tumor effect but failed to achieve pain reduction of ≥33% from baseline. The remaining 10 patients, who received ongoing anticancer treatment, showed no antitumor effects.

## Conclusion

In this open-label prospective study, the combined use of low-dose methadone and ongoing opioid treatment appeared to be safe, and reduced cancer pain that did not respond to conventional opioid therapy. Further research is needed to investigate the efficacy of the combined use of low-dose methadone and ongoing opioids, using appropriately designed randomized controlled trials.

## Data Availability

The data underlying this article will be shared on reasonable request to the corresponding author.

## References

[1] Fürst P , Lundström S, Klepstad P, Strang P. The use of low-dose methadone as add-on to regular opioid therapy in cancer-related pain at end of life: a national Swedish survey in specialized palliative care. J Palliat Med. 2020;23:226-232. 10.1089/jpm.2019.0253 31436477

[2] Wallace E , Ridley J, Bryson J, et al Addition of methadone to another opioid in the management of moderate to severe cancer pain: a case series. J Palliat Med. 2013;16:305-309. 10.1089/jpm.2012.0335 23391350

[3] Courtemanche F , Dao D, Gagné F, et al Methadone as a coanalgesic for palliative care cancer patients. J Palliat Med. 2016;19:972-978. 10.1089/jpm.2015.0525 27399839

[4] Porta-Sales J , Garzón-Rodríguez C, Villavicencio-Chávez C, et al Efficacy and safety of methadone as a second-line opioid for cancer pain in an outpatient clinic: a prospective open-label study. Oncologist. 2016;21:981-987. 10.1634/theoncologist.2015-0503 27306912 PMC4978552

[5] Kim HJ , Kim YS, Park SH. Opioid rotation versus combination for cancer patients with chronic uncontrolled pain: a randomized study. BMC Palliat Care. 2015;14:41. 10.1186/s12904-015-0038-7 26377913 PMC4572447

[6] Corli O , Roberto A, Corsi N, et al Opioid switching and variability in response in pain cancer patients. Support Care Cancer. 2019;27:2321-2327. 10.1007/s00520-018-4485-6 30357556

[7] Paice JA , Bohlke K, Barton D, et al Use of opioids for adults with pain from cancer or cancer treatment: ASCO Guideline. J Clin Oncol. 2023;41:914-930. 10.1200/JCO.22.02198 36469839

[8] Fallon M , Giusti R, Aielli F, et al ESMO Guidelines Committee. Management of cancer pain in adult patients: ESMO Clinical Practice Guidelines. Ann Oncol. 2018;29:iv166-iv191. 10.1093/annonc/mdy152 30052758

[9] Mawatari H , Shinjo T, Morita T, et al Revision of pharmacological treatment recommendations for cancer pain: clinical guidelines from the Japanese Society of Palliative Medicine. J Palliat Med. 2022;25:1095-1114. 10.1089/jpm.2021.0438 35363057

[10] McLean S , Twomey F. Methods of rotation from another strong opioid to methadone for the management of cancer pain: a systematic review of the available evidence. J Pain Symptom Manage. 2015;50:248-259.e1. 10.1016/j.jpainsymman.2015.02.029 25896106

[11] McPherson ML , Walker KA, Davis MP, et al Safe and appropriate use of methadone in hospice and palliative care: expert consensus white paper. J Pain Symptom Manage. 2019;57:635-645.e4. 10.1016/j.jpainsymman.2018.12.001 30578934

[12] Snijders RAH , Brom L, Theunissen M, van den Beuken-van Everdingen MHJ. Update on prevalence of pain in patients with cancer 2022: a systematic literature review and meta-analysis. Cancers (Basel). 2023;15:591. 36765547 10.3390/cancers15030591 PMC9913127

[13] Wiffen PJ , Wee B, Moore RA. Oral morphine for cancer pain. Cochrane Database Syst Rev. 2016;4:Cd003868. 10.1002/14651858.CD003868.pub4 27105021 PMC6540940

[14] Schmidt-Hansen M , Bennett MI, Arnold S, et al Oxycodone for cancer-related pain. Cochrane Database Syst Rev. 2022;6:Cd003870. 10.1002/14651858.CD003870.pub7 25723351

[15] Li Y , Ma J, Lu G, et al Hydromorphone for cancer pain. Cochrane Database Syst Rev. 2021;8:CD011108. 10.1002/14651858.CD011108.pub3 34350974 PMC8406835

[16] Hadley G , Derry S, Moore RA, Wiffen PJ. Transdermal fentanyl for cancer pain. Cochrane Database Syst Rev. 2013;2013:CD010270. 10.1002/14651858.CD010270.pub2 24096644 PMC6517042

[17] Quigley C. Opioid switching to improve pain relief and drug tolerability. Cochrane Database Syst Rev. 2004;3:CD004847. 10.1002/14651858.CD004847 15266542

[18] Kane CM , Mulvey MR, Wright S, et al Opioids combined with antidepressants or antiepileptic drugs for cancer pain: systematic review and meta-analysis. Palliat Med. 2018;32:276-286. 10.1177/0269216317711826 28604172

[19] Davis MP , Walsh D. Methadone for relief of cancer pain: a review of pharmacokinetics, pharmacodynamics, drug interactions and protocols of administration. Support Care Cancer. 2001;9:73-83. 10.1007/s005200000180 11305074

[20] Miyaji T , Iioka Y, Kuroda Y, et al Japanese translation and linguistic validation of the US National Cancer Institute’s Patient-Reported Outcomes version of the Common Terminology Criteria for Adverse Events (PRO-CTCAE). J Patient Rep Outcomes. 2017;1:8. 10.1186/s41687-017-0012-7 29757296 PMC5934908

[21] Farrar JT , Portenoy RK, Berlin JA, et al Defining the clinically important difference in pain outcome measures. Pain. 2000;88:287-294. 10.1016/S0304-3959(00)00339-0 11068116

[22] Reddy S , Hui D, El Osta B, et al The effect of oral methadone on the QTc interval in advanced cancer patients: a prospective pilot study. J Palliat Med. 2010;13:33-38. 10.1089/jpm.2009.0184 19824814 PMC2939847

